# Test–retest stability, convergent validity, and sensitivity to change for the Goal‐Based Outcome tool for adolescents: Analysis of data from a randomized controlled trial

**DOI:** 10.1002/jclp.23422

**Published:** 2022-08-17

**Authors:** Charlie Duncan, Mick Cooper, David Saxon

**Affiliations:** ^1^ Department of Psychology University of Roehampton London UK; ^2^ Department of Research British Association for Counselling and Psychotherapy Lutterworth UK; ^3^ Department of Psychology University of Sheffield Sheffield UK

**Keywords:** GBO tool, goals, psychometrics, school‐based counseling, young people

## Abstract

**Objective(s):**

To examine the psychometric properties of the idiographic Goal‐Based Outcome (GBO) tool for young people: test–retest stability, convergent validity, and sensitivity to an intervention.

**Methods:**

This measure validation study used data from a randomized controlled trial of school‐based humanistic counseling. We used multilevel analyses to assess test–retest stability, convergent validity of the GBO tool against nomothetic measures of mental wellbeing, and sensitivity to an intervention.

**Results:**

The GBO tool showed acceptable stability over a 6–24 week period; moderate convergent validity with nomothetic measures of mental well‐being, self‐esteem, and depression; and greater sensitivity to an intervention than a measure of psychological distress.

**Conclusions:**

The GBO tool shows evidence of having acceptable psychometric properties and is suitable for monitoring change on individual goals. It may also have the capacity to function as a population‐level indicator of outcomes in conjunction with the use of other measures of mental health and wellbeing.

## 1 INTRODUCTION

1.1

Routine outcome monitoring is the regular “measuring and tracking [of] client progress with standardized self‐report scales throughout the course of treatment” (Lambert et al., 2018, p. 521). Self‐report scales can be either “nomothetic” or “idiographic,” with nomothetic measures being made up of predetermined and predefined items, while idiographic measures allow for personalization within a standardized format (Lloyd et al., 2019). Personalized, or idiographic, measures (*idiographic patient‐reported outcome measures [I‐PROMs]*) enable clients to set their own focus for therapy and then rate their progress against this.

Nomothetic measures are, due to their predefined nature, often simpler and quicker for individuals to complete, as well as being easier to interpret (Cox & Klinger, 2021). However, they also assume a shared understanding and interpretation of the meaning that individuals assign to items (Lloyd et al., 2019). As Sales et al. (2022) write, “nomothetic PROMs are grounded in the positivist assumptions of classical test theory… that a ‘true’ score exists, along a latent, objectively ‘real’ dimension” (p. 4). Conversely, the strength of idiographic measures lies in their ability to provide clinicians and researchers with the capacity to focus on the individual and their needs, wants, and aspirations. In this respect, I‐PROMs are more consistent with a constructivist epistemology, whereby an individual's goals or problems are understood in terms of their own lived experiences (Sales et al., 2022). However, idiographic measures have been criticized for not being able to operate as population‐level indicators of outcomes due to the individualized nature of the indicators set. This often makes it difficult to compare outcomes across clients, interventions, and clinical settings (Elliott et al., 2016).

Two principal measurement models underlie psychological measures: *formative* and *reflective* (Sales et al., 2022). Formative models assume that observed indicators cause the hypothesized construct: For instance, education and income levels, together, might determine socioeconomic status. Reflective measures, in comparison, assume the opposite: that the hypothesized, or latent, construct causes—or is reflected in—the observed indicators: for instance, items such as “feeling down” or “sleeping poorly” might reflect an underlying construct of “depression” (Sales et al., 2022). Sales et al. (2022) propose that I‐PROMs, such as the Goal Based Outcome (GBO) tool (Law, 2011, 2019; Law & Jacob, 2013), can be either reflective, formative, or a mixture of the two. Hence, statistical techniques that are applicable to both models should be utilized. They state that test–retest reliability can be applied to both measurement models, but that either multilevel modeling or bivariate correlations should be used. Multilevel modeling is recommended over bivariate correlations where it cannot be assumed that scores and items are measuring the same construct between individuals. A similar recommendation is made for assessing convergent validity. Measures of internal consistency, such as Cronbach's *α*, are not recommended where a measure could be considered to be formative.

Idiographic measures for psychotherapy can be either *problem‐focused* (i.e., identifying difficulties or concerns that a client wants to overcome) or *goal‐focused* (i.e., identifying aims or objectives that a client want to achieve). It has been argued that the latter are supported by several additional lines of psychological and psychotherapeutic research (Lloyd et al., 2019). Lloyd et al. (2019) identified nine goal‐focused measures that had been used in psychotherapy, with some evidence of psychometric reliability and/or validity. Of these, just one had been used with children and young people: the GBO tool.

The GBO tool allows clients, parents/carers, and/or clinicians to set up to three goals for psychotherapy and then to rate progress on a 0 (*not met at all*) to 10 (*fully met*) scale. This can be either at every session, periodically throughout the therapeutic relationship, or at the beginning and end of psychotherapy (Law, 2011). The GBO tool has been used across a range of settings, including statutory Child and Adolescent Mental Health Services (CAMHS)—the English National Health Services for assessing and treating children and young people with mental health, emotional, and behavioral issues—and school‐based counseling services in the United Kingdom (Law & Wolpert, 2014; Rupani et al., 2014). The GBO tool has been a recommended measure for England's Children and Young People's Improving Access to Psychological Therapies (CYP IAPT) services since CYP IAPT's inception in 2011. It is also a core measure in the more recent workforce development roles of Children's Wellbeing Practitioners and the Educational Mental Health Practitioners, deriving from the UK Government Green Paper of 2017 (Department of Health and Department for Education, 2017). The GBO tool is licensed and built into the Mental Health Services Data Set and is one of NHS England's key metrics for children and young people's mental health (CYPMH) services (NHS England, 2018), highlighting its current use as a population‐level indicator of outcomes.

Despite its widespread use, psychometric data on the GBO tool remains sparse. In respect to convergent validity, preliminary evidence from CAMHS (Wolpert et al., 2012) has found that change scores on the clinician‐, parent‐, and child‐rated GBO tool correlate moderately (*r* = 0.4) with change on the clinician‐rated Child Global Assessment Scale (Shaffer et al., 1983). However, no correlation with change on the child‐rated Strengths and Difficulties Questionnaire (SDQ, Goodman, 1997) was found (Wolpert et al., 2012). In terms of sensitivity to change, Edbrooke‐Childs et al. (2015) found that the clinician, parent, and child‐rated GBO tool showed larger pre–post‐treatment effect sizes than the parent‐rated SDQ (Goodman, 1997) and the clinician‐rated CGAS in CAMHS. The authors concluded that the GBO tool may be a more sensitive measure of individual change than standardized nomothetic measures. However, it is important to note two things about this study: First, it is not possible to determine sensitivity to an intervention, given that it utilized a naturalistic dataset whereby there was no non‐intervention control group to draw comparisons with, and second, that the nomothetic measures were not self‐report and so it is difficult to determine the extent to which they reflected change that was experienced by clients. To date, and to our knowledge, the psychometric properties of the GBO tool have only been assessed at a single level (i.e. treating all goals as independent of each other), rather than employing multilevel analysis as recommended by Sales and colleagues (2022).

The aim of this study is to extend our understanding of the psychometric properties of the GBO tool. We hope to add value to the current literature in three ways. First, we will assess, for the first time, the test–retest stability of the GBO tool across moderate (6 weeks) and longer‐term (12, 18, and 24 weeks) periods. Second, we will widen tests of convergent validity by looking at the associations between baseline, 12 weeks, and baseline‐to‐12‐week change scores on the GBO tool and a range of child‐rated outcome measures, employing multilevel techniques to account for the hierarchical nature of the data (i.e., individual goals, nested within participants, nested within schools). Third, through drawing on data from a randomized controlled trial (RCT), we will provide an assessment of the GBO tool's sensitivity to an intervention, which has not been determined previously. That is, not only will we examine whether the GBO tool shows change from Time 1 to Time 2—both absolutely and relative to other measures—but also whether it picks up greater change in an intervention condition as compared with a control condition from Time 1 to Time 2 (and, again, both absolutely and relative to other measures).

## 2 METHODS

### 2.1 Design

This measure validation study tested (a) the test–retest stability of the GBO tool over a 6‐, 12‐, 18‐, and 24‐week period, (b) the convergent validity of the GBO tool against several child‐rated measures of emotional and mental wellbeing, and (c) the sensitivity of the GBO tool, compared to a measure of psychological distress (Young Person's Clinical Outcomes in Routine Evaluation [YP‐CORE]), for identifying therapeutic effects. The study draws on data from an RCT of school‐based humanistic counseling (SBHC) against usual pastoral care (see Cooper et al., 2021).

### 2.2 Participants

The trial was conducted in 18 secondary schools across London, United Kingdom. Fourteen of the eighteen schools had their own counselor, with the remaining four schools having two counselors. To participate in the study, young people needed to be aged between 13 and 16 years old, and to be experiencing moderate to severe levels of emotional symptoms (ES), as assessed by a score of 5 or more on the ES subscale of the SDQ (see below). Potentially eligible young people were identified by the schools' existing pastoral care teams and assessed for suitability if informed consent was obtained from the young person themselves and a parent/carer.

A total of 596 young people were assessed for eligibility and, of these, 329 (55.2%) were recruited to the trial, with 167 randomly allocated (1:1) to the experimental condition (SBHC plus access to the school's usual pastoral care condition, “SBHC group”) and 162 allocated to the control condition (pastoral care as usual, alone, “PCAU group”) For further information on the randomization procedure see Cooper et al. (2021). The average number of young people recruited to the study per school was 18, with the fewest number of participants recruited from a school being 5 and the maximum being 36.

Table 1 provides an overview of the sample by gender, age, ethnicity, and whether the young person considered themselves to have a disability. Overall, the majority of participants in each condition were female, aged 13–14 years, were of White, European, and/or British ethnicity, and did not consider themselves to have a disability. There were no significant differences in the characteristics of young people allocated to either the SBHC or PCAU condition.

**Table 1:** Sample demographics

Demographic		SBHC, *n* (%)	PCAU, *n* (%)
Gender			
Male		37 (22.2)	32 (19.8)
Female		127 (76.0)	129 (79.6)
Another gender		3 (1.8)	1 (0.6)
Age			
Mean age in years (SD)		13.7 (0.8)	13.8 (0.8)
Ethnicity			
White, European, and/or British		92 (55.1)	88 (54.3)
Black, mixed ethnicity, and/or other		74 (44.3)	73 (45.1)
Missing		1 (0.6)	1 (0.6)
Disability present			
Yes		25 (15.0)	26 (16.0)
No		142 (85.0)	136 (84.0)

Abbreviations: PCAU, pastoral care as usual; SBHC, school‐based humanistic counseling.

Participants allocated to SBHC received up to 10 sessions of weekly, face‐to‐face humanistic counseling, based on a clinical practice manual (Kirkbride, 2017). Participants in the PCAU condition had access to the schools' pre‐existing services for supporting the emotional health and well‐being of young people. This can vary substantially across schools and pupils but might consist, for instance, of time with a personal tutor or meetings with a school nurse.

### 2.3 Measures

Measurement points were at baseline, 6 (mid‐point), 12 (endpoint), and 24 weeks (follow‐up) for all measures.

#### 2.3.1 GBO tool

The GBO tool allows clients, parents/carers, and/or clinicians to set up to three personal goals for psychotherapy by writing in a free‐text box. For the present study, only the child‐rated version was used. Progress towards goals is rated on a 0 (*not met at all*) to 10 (*fully met*) scale (Law, 2011).

A reliable change index (RCI) of 2.45 was previously calculated for the GBO tool (Edbrooke‐Childs et al., 2015), although this was based on data from CAMHS settings. Therefore, we calculated the RCI in the present study using the data from those in the SBHC condition, in line with the method devised by Jacobson and Truax (1991), which involves calculating the difference between a client's baseline goal progress score and 12‐week goal progress score, and dividing by the standard error (SE) of the difference between the two scores. An RCI of 2.52 was calculated for the present study, which is comparable to that calculated by Edbrooke‐Childs et al. (2015). Hence, for the present study, a change of three or more in either direction on any individual goal was considered as reliable change due to progress being rated as a whole number and therefore change only being possible as a whole integer.

In the present study, personal goals were set by the young people at baseline assessment and these same goals were rated at each timepoint. Baseline assessments were undertaken in the presence of assessors, rather than the counselor who was allocated to provide the intervention at each school. Examples of the personal goals set by the young people included “wanting to feel more confident,” “wanting to improve relationship(s) with family member(s)” and “wanting to concentrate more in school.”

#### 2.3.2 Young Person's Clinical Outcomes in Routine Evaluation

The YP‐CORE (Twigg et al., 2009) is a 10‐item self‐report measure of psychological distress which has been validated for use with young people aged 11–16. Examples of items on YP‐CORE include: “I have felt edgy or nervous” and “there's been someone I felt able to ask for help,” with the response options being “not at all,” “only occasionally,” “sometimes,” “often,” and “most or all of the time.” The measure has good levels of external validity, internal reliability, and acceptability (Twigg et al., 2009, 2016). Reliable change indices and clinical thresholds for YP‐CORE are gender‐ and age‐band specific (see Twigg et al., 2016).

Using data collected as part of the present study, the test–retest stability of YP‐CORE was calculated as *r* = 0.53, *r* = 0.62, and *r* = 0.63 at 0–6, 6–12 weeks, and 12–24 weeks respectively, indicating high levels of stability over these time periods.

#### 2.3.3 Strengths and Difficulties Questionnaire

The SDQ (Goodman, 1997) is a brief (25‐item) behavioral screening questionnaire for children and young people aged 3–16 years old, which has good levels of reliability and validity (Goodman, 2001). It has five subscales—conduct problems (CPs), ES, hyperactivity (HA), peer relationships (PP), and prosocial behavior (PS)—with a total difficulties score consisting of the combined scores of the former four subscales (SDQ‐TD). Items include: “I worry a lot,” “I have one good friend or more,” and “I am often accused of lying or cheating,” with the response options being “not true,” “somewhat true,” and “certainly true.” The self‐report version of the SDQ was utilized in the present study which can be completed by those aged between 11 and 16 years.

From the data collected as part of the present study, the test–retest stability of the SDQ‐TD was *r* = 0.65, *r* = 0.73, and *r* = 0.75 at 0–6, 6–12, and 12–24 weeks respectively, indicating high levels of stability over these time periods.

#### 2.3.4 Revised Child Anxiety and Depression Scale‐Short Version

The Revised Child Anxiety and Depression Scale‐Short Version (RCADS‐SV; Ebesutani et al., 2012) is a 25‐item self‐report tool which is designed to screen for depression and anxiety in children and young people aged 8–18 years. Items on RCADS‐SV include: “I worry what other people think of me” and “I feel worthless,” with respondents asked to select whether these occur “never,” “sometimes,” “often,” or “always.” It has two subscales—anxiety and depression—as well as a total score. RCADS‐SV has been found to have acceptable levels of reliability and validity (Ebesutani et al., 2012). The test–retest stability of the RCADS‐SV total scores in the present study was high at 0–6 (*r* = 0.71), 6–12 (*r* = 0.81), and 12–24 weeks (*r* = 0.80).

#### 2.3.5 Rosenberg Self‐Esteem Scale

The Rosenberg Self‐Esteem Scale (RSE; Rosenberg, 1965) is a 10‐item self‐report measure of self‐esteem, which includes items such as “at times I think I am no good at all” and “I am able to do things as well as most people.” Response options are “strongly agree,” “agree,” “disagree,” or “strongly disagree.” It has been shown to have good levels of test–retest reliability and concurrent, predictive, and construct validity (Rosenberg, 1965). The test–retest stability of the RSE in the present study was high at 0–6 (*r* = 0.72), 6–12 (*r* = 0.76), and 12–24 weeks (*r* = 0.81).

#### 2.3.6 Warwick–Edinburgh Mental Well‐Being Scale

The Warwick–Edinburgh Mental Well‐Being Scale (WEMWBS) (Tennant et al., 2007) is a 14‐item self‐report tool designed to monitor an individual's mental wellbeing and has been validated for use with young people from the age of 13 years (Clarke et al., 2011). Items on the WEMWBS include: “I've been feeling useful,” “I've been thinking clearly,” and “I've been feeling confident,” with respondents asked to select if they've experienced these “none of the time,” “rarely,” “some of the time,” “often,” or “all of the time” over the last 2 weeks. The measure has demonstrated good levels of reliability and validity (Clarke et al., 2011; Tennant et al., 2007). From the data collected as part of the present study, the test–retest stability of the WEMWBS was *r* = 0.56, *r* = 0.63, and *r* = 0.08 at 0–6, 6–12, and 12–24 weeks, respectively. This indicates high levels of stability over the short (6 weeks) period, but poor stability over a longer period (12 weeks).

### 2.4 Analytic strategy

Test–retest stability and convergent validity were determined through multilevel analyses. This was to account for the nested structure and non‐independence of individual goal progress scores, which were nested within individual goals (Level 1), participants (Level 2), and schools (Level 3). However, school level variability was not a significant predictor of individual goal progress scores at any timepoint—parameter estimates for the intraclass correlation coefficients (ICCs) of goal progress scores at the school level ranged from 0.002 to 0.017 at baseline, 12 weeks, and baseline‐to‐12‐week change—and therefore was not included in any models. This resulted in a two‐level analysis: individual goals (Level 1) and participants (Level 2). The parameter estimates for the ICCs of goal progress scores at the participant level ranged from 0.22 to 0.27 across all models (i.e., between 22% and 27% of the variance in goal progress scores can be explained at the participant level).

For each model, predictor variables were entered and improvement in the model in comparison to the initial model was tested using the likelihood ratio statistic (Rasbach et al., 2019) and inspection of the predictor variables coefficients and SEs. Predictor variables were entered as fixed factors and continuous variables were centered around their grand mean, to aid interpretation (Snijders & Bosker, 2011). The proportional change in total variance from the initial model to subsequent models was calculated (i.e., the amount of variance explained by the predictor variable[s]) as a decimal and then the square root was calculated to compute an equivalence to Pearson's *r* (as set out in Snijders & Bosker, 2011). In line with the suggestion by Cohen (1988), the magnitudes of the correlation coefficients were classed as weak (0.1<|*r*|<0.3), moderate (0.3<|*r*|<0.5), or strong (|*r*|0.5).

#### 2.4.1 Test–retest stability

Test–retest stability was assessed between individual goal progress scores at all timepoints: 0–6, 0–12, 0–24, 6–12, 6–24, and 12–24 weeks. For 0–6 week stability, the initial model included a random intercept with 6‐week goal progress score as the dependent variable. The baseline goal progress score was then added to the model as a predictor variable. For 12‐week stability, the initial model included a random intercept with 12‐week goal progress score as the dependent variable. Baseline and 6‐week goal progress scores were then added into the model separately. For 24‐week stability, the initial model included a random intercept, with 24‐week goal progress score as the dependent variable. Baseline, 6‐ and 12‐week goal progress scores were then added into the model separately.

#### 2.4.2 Convergent validity

Convergence was assessed between individual GBO tool scores (dependent variable) and the corresponding YP‐CORE, SDQ, WEMWBS, RSE, and RCADS‐SV scores at baseline, 12 weeks, and baseline‐to‐12‐week change, respectively. The initial model at each timepoint included a random intercept and any significant participant‐level predictors (e.g., gender, age, ethnicity, condition, and baseline goal score). Covariates of gender, age, ethnicity, condition, and measure scores were entered into the initial model as fixed factors. Measure scores were standardized based on their grand mean.

#### 2.4.3 Sensitivity to an intervention

Sensitivity to an intervention was assessed by comparing the proportion of clients showing reliable change (as determined using RCIs) on the GBO tool and the YP‐CORE. Reliable improvement on the GBO tool was determined as increasing by three or more points on any single goal set by a participant, provided no other goal had reliably deteriorated (reduced by three or more points), in line with the recommendation by Jacob et al. (2021). Hence, for the GBO tool, participants were identified as falling into one of four categories: (1) reliably improved (reliably improved on at least one personal goal but not reliably deteriorated on a single personal goal), (2) reliably deteriorated (reliably deteriorated on at least one personal goal, but not reliably improved on a single personal goal, (3) no change (a change of less than three in any direction on all personal goals set, (4) uncategorized (reliably improving on at least one personal goal, but also reliability deteriorating on at least one other personal goal).

Analyses were undertaken using the Statistical Package for the Social Sciences version 26 (IBM Corp, 2019), Stata (StataCorp, 2019), and MLwiN 3.02 (Centre for Multilevel Modelling, 2018).

### 2.5 Ethical approval

Ethical approval for this study was granted by the University Ethics Committee of the University of Roehampton (reference PSYC 16/227).

## 3 RESULTS

Table 2 provides an overview of the mean and standard deviations of individual goal progress scores at each timepoint, both overall and split by condition.

**Table 2:** Mean and standard deviations of individual goal progress scores at each timepoint, both overall and split by condition

Timepoint	Overall (*M*, SD)	PCAU (*M*, SD)	SBHC (*M*, SD)
Baseline	2.92 (1.82)	2.99 (1.85)	2.84 (1.78)
6 weeks	4.34 (2.48)	3.76 (2.37)	4.92 (2.46)
12 weeks	5.19 (2.59)	4.34 (2.43)	6.06 (2.45)
24 weeks	5.47 (2.76)	4.82 (2.70)	6.15 (2.65)

Abbreviations: PCAU, pastoral care as usual; SBHC, school‐based humanistic counseling.

### 3.1 Test–retest stability

Over the moderate term, the test–retest correlations on individual goal progress scores between baseline and 6 weeks was *r* = 0.40, and between 6 and 12 weeks it was *r* = 0.62. Over the longer term (12 weeks), the test–retest correlation on individual goal progress scores between baseline and 12 weeks was *r* = 0.31, and between 12 and 24 weeks was *r* = 0.64. Over an 18 week period (6–24 weeks), test–retest stability was *r* = 0.54, and over a 24‐week period (baseline–24 weeks) it was *r* = 0.27.

### 3.2 Convergent validity

#### 3.2.1 Baseline scores

Table 3 presents the multilevel estimates for the models predicting baseline individual goal progress scores. Gender, age, condition, and ethnicity were not statistically significant predictor variables (*p* > 0.05). Hence, the null model included just the random intercept. Baseline scores for each measure and subscales were then added into the model one‐by‐one.

**Table 3:** Multilevel estimates for models predicting baseline GBO tool scores convergence with other self‐report measures baseline scores

Model and variables	Coefficient (predictor variable[s])	SE (predictor variable[s])	Level 1 (goal) intercept variance (SE)	Level 2 (participant) intercept variance (SE)	−2* LL	Diff −2* LL (*df*)	Proportional change in total variance	*r*
Null model: Intercept			2.57 (0.15)	0.74 (0.14)	3644.33			
Model 1: Intercept, YP‐CORE	−0.06**	0.010	2.50 (0.15)	0.66 (0.13)	3607.18	−37.15 (1)	−4.50%	0.21
Model 2: Intercept, WEMWBS	0.07**	0.007	2.46 (0.14)	0.54 (0.12)	3568.31	−76.02 (1)	−9.40%	0.31
Model 3: Intercept, RSE	0.11**	0.013	2.47 (0.14)	0.55 (0.12)	3574.99	−69.34 (1)	−8.64%	0.29
Model 4: Intercept, RCADS‐SV total	−0.04**	0.006	2.49 (0.15)	0.58 (0.13)	3588.69	−55.64 (1)	−7.04%	0.27
Model 5: Intercept, RCADS‐SV anxiety subscale	−0.05**	0.009	2.52 (0.15)	0.65 (0.13)	3611.50	−32.83 (1)	−4.20%	0.21
Model 6: Intercept, RCADS‐SV depression subscale	−0.10**	0.012	2.48 (0.14)	0.57 (0.13)	3582.40	−61.93 (1)	−7.77%	0.28
Model 7: Intercept, SDQ total difficulties	−0.09**	0.014	2.52 (0.15)	0.63 (0.13)	3607.07	−37.26 (1)	−4.87%	0.22
Model 8: Intercept, SDQ PS subscale	0.05	0.036	2.57 (0.15)	0.73 (0.14)	3642.50	−1.83 (1)	−0.27%	0.05
Model 9: Intercept, SDQ HA subscale	−0.10**	0.028	2.59 (0.15)	0.65 (0.14)	3631.67	−12.66 (1)	−2.24%	0.15
Model 10: Intercept, SDQ ES subscale	−0.26**	0.042	2.47 (0.14)	0.69 (0.13)	3605.94	−38.39 (1)	−4.32%	0.21
Model 11: Intercept, SDQ CP subscale	−0.10**	0.038	2.54 (0.15)	0.74 (0.14)	3636.88	−7.45 (1)	−0.79%	0.09
Model 12: Intercept, SDQ PP subscale	−0.10**	0.034	2.55 (0.15)	0.72 (0.14)	3635.95	−8.38 (1)	−1.03%	0.10
Model 13: Intercept, SDQ externalizing problems subscale	−0.08**	0.019	2.57 (0.15)	0.67 (0.14)	3629.72	−14.61 (1)	−2.21%	0.15
Model 14: Intercept SDQ internalizing problems subscale	−0.14**	0.024	2.50 (0.15)	0.69 (0.13)	3612.27	−32.06	−3.78%	0.19

Abbreviation: CP, conduct problem; Diff, difference; ES, emotional symptom; GBO, Goal‐Based Outcome; HA, hyperactivity; LL, log‐likelihood; PP, peer relationships; PS, prosocial behavior; RCADS‐SV, Revised Child Anxiety and Depression Scale‐Short Version; RSE, Rosenberg Self‐Esteem Scale; SDQ, Strengths and Difficulties Questionnaire; WEMWBS, Warwick–Edinburgh Mental Well‐Being Scale; YP‐CORE, Young Person's Clinical Outcomes in Routine Evaluation.

* *p* < 0.05

** *p* < 0.01.

All models, except that of the SDQ PS subscale baseline score (*p* = 0.18), significantly improved on the null model. Of these, WEMWBS baseline scores were the strongest predictor of baseline goal progress scores (*r* = 0.31), followed by RSE baseline scores (*r* = 0.29), RCADS‐SV depression subscale baseline scores (*r* = 0.28) and RCADS‐SV total scores at baseline (*r* = 0.27). The weakest predictors of baseline goal progress scores were SDQ CP subscale baseline scores (*r* = 0.09), SDQ PP subscale baseline scores (*r* = 0.10), SDQ HA subscale scores (*r* = 0.15) and SDQ externalizing problems baseline scores (*r* = 0.15).

#### 3.2.2 12‐Week scores

Supporting Information Appendix: Table S1 presents the multilevel estimates for the models predicting 12‐week individual goal progress scores. The null model included the covariates of age, condition, and baseline goal progress score and was a significantly better fit than the model without these predictors and therefore this was used as the comparator model for the 12‐week analyses. Ethnicity and gender were not significant predictors and so were not included in the models. Twelve‐week scores for each other self‐report measure and their subscales were then added into the model one‐by‐one.

All models were a significantly better fit at the *p* < 0.01 level than the null model. Of these, WEMWBS scores at 12 weeks were the strongest predictor of 12 week goal progress scores (*r* = 0.41), followed by YP‐CORE scores at 12 weeks (*r* = 0.40), and RCADS‐SV depression subscale scores at 12 weeks (*r* = 0.37). The weakest predictors of 12 week goal progress scores were SDQ PS subscale 12 week scores (*r* = 0.11), SDQ PP subscale 12 week scores (*r* = 0.14), and SDQ CP subscale 12 week scores (*r* = 0.17).

#### 3.2.3 Baseline‐to‐12‐week change

Supporting Information Appendix: Table S2 presents the multilevel estimates for the models predicting baseline‐to‐12 week individual goal progress change scores. The null model included the covariates of age, condition, and baseline goal progress score and was a significantly better fit than the model without these predictors and therefore this was used as the comparator model for the baseline‐to‐12‐week change analyses. Ethnicity and gender were not significant predictors and so were not included in the models. Baseline‐to‐12‐week change scores for each other measure and their subscales were then added into the model one‐by‐one.

All models, except that of the SDQ PS subscale baseline score (*p* = 0.66), significantly improved on the null model. Models for all other measures were a significantly better fit than the null model at the *p* < 0.01 level. Of these models, RCADS‐SV total change scores weeks were the strongest predictor of baseline‐to‐12‐week change on goal progress scores (*r* = 0.38), followed by RCADS‐SV depression subscale change scores (*r* = 0.37), and WEMWBS change scores (*r* = 0.34). Although still statistically significant, the weakest predictors of baseline‐to‐12‐week goal progress change scores were SDQ CP subscale change scores (*r* = 0.13), SDQ PP subscale change scores (*r* = 0.13), and SDQ HA subscale change scores (*r* = 0.17).

### 3.3 Sensitivity to an intervention

Table 4 details the proportion of participants showing reliable improvement, reliable deterioration, no reliable change, and inconclusive change on the GBO tool and YP‐CORE between baseline and 12 weeks. Of all participants (*n* = 329), 311 (94.5%) rated at least one personal goal at baseline and 12 weeks and were included in these analyses.

**Table 4:** Proportion of clients showing reliable improvement, reliable deterioration, and no reliable change on the GBO tool and YP‐CORE, both overall and split by condition

		Condition		
Measure	Change	Overall, *n* (%)	PCAU, *n* (%)	SBHC, *n* (%)
GBO tool (overall *n* = 311; PCAU *n* = 158; SBHC *n* = 153)	No reliable change	90 (28.9)	67 (42.4)	23 (15.0)
	Reliable improvement	199 (64.0)	75 (47.5)	124 (81.0)
	Reliable deterioration	16 (5.1)	12 (7.6)	4 (2.6)
	Inconclusive	6 (1.9)	4 (2.5)	2 (1.3)
YP‐CORE (*n* = 325; PCAU *n* = 161; SBHC *n* = 164)	No reliable change	219 (67.4)	115 (71.4)	104 (63.4)
	Reliable improvement	87 (26.8)	35 (21.7)	52 (31.7)
	Reliable deterioration	19 (5.8)	11 (6.8)	8 (4.9)

Abbreviations: GBO, Goal‐Based Outcome; PCAU, pastoral care as usual; SBHC, school‐based humanistic counseling; YP‐CORE, Young Person's Clinical Outcomes in Routine Evaluation.

Irrespective of condition, 199 (64.0%) of the 311 participants showed reliable improvement on the GBO tool between baseline and 12 weeks; 16 (5.1%) showed reliable deterioration on the GBO tool, 90 (28.9%) showed no reliable change on the GBO tool, and the remaining 6 (1.9%) were inconclusive. In comparison, where reliable change on the YP‐CORE could be calculated for participants with complete gender, age, baseline YP‐CORE, and 12‐week YP‐CORE score (*n* = 325, 98.8%), 87 (26.8%) showed reliable improvement, 19 (5.8%) showed reliable deterioration, and 219 (67.4%) showed no reliable change. Of those who showed reliable improvement on the YP‐CORE (*n* = 87), 74 (85.1%) also showed reliable improvement on the GBO tool.

In the SBHC condition (*n* = 153), 124 (81.0%) participants showed reliable improvement on the GBO tool, 4 (2.6%) showed reliable deterioration, 23 (15.0%) showed no reliable change, and 2 (1.3%) were uncategorized. This compared to 52 (31.7%) showing reliable improvement on the YP‐CORE, 8 (4.9%) showing reliable deterioration, and 104 (63.4%) showing no reliable change. Of those who showed reliable improvement on the YP‐CORE, 49 (94.2%) also showed reliable improvement on the GBO tool.

In the PCAU condition (*n* = 158), 75 participants (47.5%) showed reliable improvement on the GBO tool, 12 (7.6%) showed reliable deterioration, 67 (42.4%) showed no reliable change, and 4 (2.5%) were uncategorized. Conversely, 35 (21.6%) showed reliable improvement on the YP‐CORE, 11 (6.8%) showed reliable deterioration and the remaining 115 (71.4%) showed no reliable change on the YP‐CORE. Of those who showed reliable improvement on the YP‐CORE, 25 (71.4%) also showed reliable improvement on the GBO tool.

## 4 DISCUSSION

Our findings provide new and more accurate information on the psychometric properties of the GBO tool, using multilevel analysis techniques. This is the first study to explore the test–retest stability of the GBO tool and the convergent validity of the GBO tool with other child‐rated outcome measures in this way. Furthermore, we have assessed the sensitivity to an intervention of the GBO tool in an intervention group compared to a control group, which again, has not been undertaken previously. Together, our findings contribute to the body of evidence supporting the current use of the GBO tool as an individual‐ and population‐level indicator of outcomes.

Most importantly, this study significantly develops our understanding of the sensitivity of the GBO tool to an intervention, which has not been evidenced previously. In contrast to previous research, the present study investigated the sensitivity of the GBO tool, against other self‐report measures, in an intervention group compared to a control group. In line with findings from Edbrooke‐Childs et al. (2015), we determined that the GBO tool was a much more sensitive measure to individual change than standardized measures. Indeed, in the present study, 94.2% of those young people who showed reliable improvement on the YP‐CORE in the intervention condition, also showed reliable improvement on the GBO tool, which highlights the ability of the GBO tool to pick up on change identified on other measures. However, and in addition to this, over twice as many participants, across both conditions, showed reliable improvement on the GBO tool compared to the YP‐CORE. This may be due to the flexibility that idiographic measures allow for clients to highlight their priorities for change, which may not always be captured in nomothetic measures (Ashworth et al., 2007).

This indicates that the GBO tool can detect intervention effects that other measures cannot, and further suggests that it should be one of the tools used to evaluate outcomes in psychotherapy for young people, to ensure that the change experienced by young people receiving such an intervention is captured. However, it is important to note that convergence between individual goal progress scores and YP‐CORE were moderate at all timepoints, so it may not be surprising that some change captured on the GBO tool is not picked up on YP‐CORE. This comparison of the proportion of young people showing reliable change on the GBO tool compared to other measures has not been demonstrated in previous research and therefore significantly extends the existing literature. Future research should look to further build on this by assessing the proportion of reliable change on the GBO tool compared to other measures for which RCIs are available. Considered together, it appears likely that the GBO tool measures change which is not detected on other outcome measures, and should be used in addition to existing outcome measures. Furthermore, the ability of the GBO tool to effectively differentiate between those receiving an intervention and those who are not, provides some evidence to support its use as a population‐level indicator of outcomes.

With respect to test–retest stability, we found moderate correlations over the medium (6–12 weeks) term, and weak‐to‐moderate correlations over the longer term (18–24 weeks). While these correlations are acceptable, they are generally not as strong as the test–retest stability of the nomothetic measures utilized as part of the current study, which tended to be around *r* = 0.60 or above. However, it seems appropriate to suggest that the GBO tool could be considered stable over a moderate to longer term time period, which has not been demonstrated in previous research. Future research which looks at the stability of the measure over a shorter period, for example, 1–2 weeks intervals, would be welcomed as this is a more appropriate time period for assessing test–retest stability (Streiner et al., 2015) than that used in the present study, and it would help to determine the extent to which it can be considered a reliable session‐by‐session measure.

Our research also shows that GBO tool scores—rated specifically from the perspective of the service user (young person)—correlate with other child‐rated indicators of psychological wellbeing and distress. Generally, at all timepoints, individual goal progress scores were most strongly correlated with scores on WEMWBS and RCADS‐SV (total scores and depression subscale scores), although these correlations were moderate. In line with previous findings (Wolpert et al., 2012), correlations between the GBO tool and the child‐reported SDQ were weakest, suggesting that goal progress is less likely to be related to changes in prosocial behavior, emotional symptoms, conduct problems, hyperactivity, and peer problems. It should be noted that previous research which has investigated the psychometric properties of the GBO tool (e.g., Edbrooke‐Childs et al., 2015; Wolpert et al., 2012) have not utilized appropriate multilevel techniques, and therefore have not controlled for clustering within data (i.e., at the client level) which may have led to overestimations of reliability and convergent validity.

### 4.1 Limitations

All measures were self‐reported by the young people and therefore it is possible that correlations between measures have been overestimated due to common method variance. Second, the frequency with which the GBO tool was collected (at baseline, 6, 12, and 24 weeks), makes it difficult to determine the test–retest stability of the tool on a short‐term (i.e., weekly) basis and hence its appropriateness as a session‐by‐session measure. Furthermore, the present study was unable to compare the proportion of participants showing reliable change on the GBO tool with any measures other than the YP‐CORE, as RCIs have not been calculated for the other measures included in this study.

### 4.2 Implications for practice

Our findings provide robust evidence for the use of the GBO tool as a routinely used outcome measure for monitoring individual change in young people. In addition, given the convergence of the GBO tool with some nomothetic routine outcome measures (N‐PROMs), as well as its ability to detect difference in change between an intervention and control group, our study provides new evidence to support its use as a population‐level indicator of outcomes, in addition to other self‐report measures.

### 1 PEER REVIEW

The peer review history for this article is available at https://publons.com/publon/10.1002/jclp.23422

## Supporting information

Supporting information.

## Data Availability

The data that support the findings of this study are openly available in ReShare at https://reshare.ukdataservice.ac.uk/853764/, reference number 10.5255/UKDA‐SN‐853764
