# Supplementary material for: Test–retest stability, convergent validity, and sensitivity to change for the Goal‐Based Outcome tool for adolescents: Analysis of data from a randomized controlled trial
Source: J Clin Psychol. 2022 Aug 17;79(3):683–96. doi: 10.1002/jclp.23422 (PMC10087879; doi:10.1002/jclp.23422)
Supplement: Supplementary file 1 — Supporting information. [file JCLP-79-683-s001.docx]

**Supplementary Material**

**Table SM1**

Multilevel estimates for models predicting 12 week GBO tool scores convergence with other self-report measures 12 week scores. Models 1 – 14 only include coefficient and standard error (SE) values for the self-report measures 12 week scores

| Model and variables | Coefficient (predictor variable(s)) | SE (predictor variable(s)) | Level 1 (goal) intercept variance (SE) | Level 2 (participant) intercept variance (SE) | -2*LL | Diff -2*LL (df) | Proportional change in total variance | *r* |
| --- | --- | --- | --- | --- | --- | --- | --- | --- |
| Null model  Intercept, covariates (condition, age, baseline goal score) | Condition: 1.79**  Age: -.44**  Baseline goal score: .40** | Condition: .167  Age: .110  Baseline goal score: .042 | 3.72 (.23) | 1.41 (.24) | 3814.41 |  |  |  |
| Model 1  Intercept, covariates, YP-CORE | -.12** | .010 | 3.47 (.21) | .82 (.18) | 3687.84 | -126.57 (1) | -16.34% | .40 |
| Model 2  Intercept, covariates, WEMWBS | .09** | .008 | 3.46 (.21) | .80 (.18) | 3670.19 | -144.22 (1) | -16.89% | .41 |
| Model 3  Intercept, covariates, RSE | .13** | .015 | 3.59 (.22) | 1.02 (.20) | 3727.14 | -87.27 (1) | -10.22% | .32 |
| Model 4  Intercept, covariates, RCADS-SV total | -.06** | .006 | 3.57 (.21) | .93 (.20) | 3724.60 | -89.81 (1) | -12.23% | .35 |
| Model 5  Intercept, covariates, RCADS-SV anxiety subscale | -.08** | .011 | 3.64 (.22) | 1.11 (.21) | 3762.03 | -52.38 (1) | -7.47% | .27 |
| Model 6  Intercept, covariates, RCADS-SV depression subscale | -.14** | .013 | 3.53 (.21) | .89 (.19) | 3710.73 | -103.68 (1) | -13.79% | .37 |
| Model 7  Intercept, covariates, SDQ total difficulties | -.13** | .015 | 3.54 (.21) | 1.06 (.20) | 3735.85 | -78.56 (1) | -10.36% | .32 |
| Model 8  Intercept, covariates, SDQ PS subscale | .13** | .044 | 3.71 (.22) | 1.35 (.23) | 3806.10 | -8.31 (1) | -1.29% | .11 |
| Model 9  Intercept, covariates, SDQ HA subscale | -.21** | .035 | 3.67 (.22) | 1.21 (.22) | 3781.08 | -33.33 (1) | -4.84% | .22 |
| Model 10  Intercept, covariates, SDQ ES subscale | -.32** | .035 | 3.50 (.21) | 1.12 (.21) | 3735.96 | -78.45 (1) | -9.93% | .32 |
| Model 11  Intercept, covariates, SDQ CP subscale | -.23** | .053 | 3.71 (.22) | 1.27 (.23) | 3796.34 | -18.07 (1) | -2.89% | .17 |
| Model 12  Intercept, covariates, SDQ PP subscale | -.19** | .047 | 3.66 (.22) | 1.36 (.23) | 3798.16 | -16.25 (1) | -1.99% | .14 |
| Model 13  Intercept, covariates, SDQ externalizing problems subscale | -.16** | .025 | 3.66 (.22) | 1.18 (.22) | 3775.99 | -38.42 (1) | -5.58% | .24 |
| Model 14  Intercept, covariates, SDQ internalizing problems subscale | -.20** | .024 | 3.53 (.21) | 1.17 (.21) | 3748.53 | -65.88 (1) | -8.37% | .29 |

**Table SM2**

Multilevel estimates for models predicting baseline-to-12 week GBO tool change scores convergence with other self-report measures baseline-to-12 week change scores. Models 1 – 14 only include coefficient and standard error (SE) values for the self-report measures baseline-to-12 week change scores

| Model and variables | Coefficient (predictor variable(s)) | SE (predictor variable(s)) | Level 1 (goal) intercept variance (SE) | Level 2 (participant) intercept variance (SE) | -2*LL | Diff -2*LL (df) | Proportional change in total variance | *r* |
| --- | --- | --- | --- | --- | --- | --- | --- | --- |
| Null model  Intercept, covariates (condition, age, baseline goal score) | Condition: 1.79**  Age: -.44**  Baseline goal score: -.60** | Condition: .167  Age: .110  Baseline goal score: .042 | 3.72 (.23) | 1.41 (.24) | 3814.41 |  |  |  |
| Model 1  Intercept, covariates, YP-CORE | .09** | .010 | 3.54 (.21) | 1.09 (.21) | 3739.78 | -74.63 (1) | -9.77% | .31 |
| Model 2  Intercept, covariates, WEMWBS | .08** | .008 | 3.54 (.21) | .98 (.20) | 3712.00 | -102.41 (1) | -11.90% | .34 |
| Model 3  Intercept, covariates, RSE | .14** | .017 | 3.61 (.22) | 1.07 (.21) | 3738.17 | -76.24 (1) | -8.82% | .30 |
| Model 4  Intercept, covariates, RCADS-SV total | -.08** | .008 | 3.54 (.21) | .86 (.19) | 3709.24 | -105.17 (1) | -14.12% | .38 |
| Model 5  Intercept, covariates, RCADS-SV anxiety subscale | -.12** | .013 | 3.59 (.22) | 1.01 (.20) | 3739.51 | -74.90 (1) | -10.32% | .32 |
| Model 6  Intercept, covariates, RCADS-SV depression subscale | -.16** | .015 | 3.54 (.21) | .88 (.19) | 3712.26 | -102.15 (1) | -13.71% | .37 |
| Model 7  Intercept, covariates, SDQ total difficulties | -.15** | .018 | 3.55 (.21) | 1.12 (.21) | 3746.87 | -67.54 (1) | -8.84% | .30 |
| Model 8  Intercept, covariates, SDQ PS subscale | .03 | .056 | 3.73 (.20) | 1.40 (.24) | 3814.22 | -.19 (1) | -.08% | .03 |
| Model 9  Intercept, covariates, SDQ HA subscale | -.21** | .045 | 3.68 (.22) | 1.29 (.23) | 3793.49 | -20.92 (1) | -3.02% | .17 |
| Model 10  Intercept, covariates, SDQ ES subscale | -.33** | .039 | 3.52 (.21) | 1.14 (.21) | 3744.01 | -70.40 (1) | -8.97% | .30 |
| Model 11  Intercept, covariates, SDQ CP subscale | -.20** | .061 | 3.72 (.22) | 1.32 (.23) | 3803.58 | -10.83 (1) | -1.81% | .13 |
| Model 12  Intercept, covariates, SDQ PP subscale | -.20** | .052 | 3.67 (.22) | 1.37 (.23) | 3800.54 | -13.87 (1) | -1.72% | .13 |
| Model 13  Intercept, covariates, SDQ externalizing problems subscale | -.19** | .034 | 3.68 (.22) | 1.23 (.22) | 3785.63 | -28.78 (1) | -4.29% | .21 |
| Model 14  Intercept, covariates, SDQ internalizing problems subscale | -.22** | .027 | 3.53 (.21) | 1.19 (.21) | 3752.05 | -62.36 (1) | -7.84% | .28 |
